# Estimating renal function in old people: an in-depth review

**DOI:** 10.1007/s11255-017-1682-z

**Published:** 2017-09-15

**Authors:** Maharajan Raman, Rachel J. Middleton, Philip A. Kalra, Darren Green

**Affiliations:** 1 Vascular Research Group, Department of Renal Medicine, Salford Royal NHS Foundation Trust, Stott Lane, Salford, M6 8HD UK; 2 Faculty of Biology, Medicine and Health, University of Manchester, Manchester, UK

**Keywords:** Old people, Estimation of GFR, Renal function

## Abstract

Estimates of glomerular filtration rate (eGFR) should provide accurate measure of an individual’s kidney function because important clinical decisions such as timing of renal replacement therapy and drug dosing may be dependent on eGFR. Formulae from which eGFR is derived are generally based on serum creatinine measurement, such as Cockcroft–Gault, MDRD and CKD-EPI. More recently, calculation of eGFR using other laboratory biomarkers such as cystatin C has emerged with apparent greater accuracy. In old people, there is age-related physiological change in the kidney, which could lead to reduced GFR. Likewise, physiological changes in body composition that occur with the ageing process impede the use of a single creatinine-based calculation of eGFR across all adult age groups. Studies have shown differences in the prevalence of CKD based on the type of equation used to estimate GFR. This review discusses the evolution of eGFR calculations and the relative accuracy of such equations in older population.

## Introduction

Accurate measure of renal function is a vital part of day-to-day safe medical care. For example, overestimation of glomerular filtration rate (GFR) may lead to drug toxicity, and underestimation of GFR may lead to sub-therapeutic drug levels. Potentially, life-changing clinical decisions such as when to start dialysis are also partly based on GFR measurement. Some guidelines recommended that planning for patients’ future dialysis should begin when their estimated GFR (eGFR) is <30 ml/min/1.73 m^2^ and declining. Dialysis usually begins when patients become symptomatic with an eGFR of <15 ml/min/1.73 m^2^.

GFR calculated by measuring the clearance of inulin by the kidney is considered “Gold standard”; precise measurement of GFR is possible but expensive, time-consuming and impractical. For this reason, there has been a constant strive to develop an equation which can estimate GFR reliably from blood and urine biochemical markers such as creatinine. These are rapid and cost-effective. However, with age there is a change in both renal physiology and muscle mass, both of which may affect eGFR calculation [1, 2]. This means that eGFR calculation may be less reliable in older patients and can therefore potentially adversely affect their clinical care. The change in renal function related to age was described as early as 1950 [3]. This study evaluated longitudinal rate of change of renal function using inulin clearance (described below) in patients not known to have renal disease. Between the ages of 20 and 90 years, the mean inulin clearance dropped from 122.8 to 65.3 ml/min/1.73 m^2^.

Similarly, the described incident rate and prevalence of chronic kidney disease (CKD) can also vary with the method of estimation of GFR. In a cross-sectional study of 9931 institutionalised individuals aged >65 years, Garg et al. [4] compared the prevalence of CKD when defined by different equations. Here, among individuals with a predicted eGFR of <30 ml/min by the Cockcroft–Gault equation [5], 14.7% had a corresponding eGFR of >60 ml/min/1.73 m^2^ and therefore no CKD when using the four-variable MDRD equation [6]. Furthermore, the prevalence of GFR of <30 ml/min using the Cockcroft–Gault equation was far higher than with MDRD in both men and women: 10.3% (95% confidence intervals 9.2–11.5%) versus 3.5% (2.8–4.2%) in men, and 23.3% (22.4–24.3%) versus 4.0% (3.6–4.5) in women.

## Gold standard measurement

The clearance of inulin by the kidney is considered a gold standard method to calculate GFR because inulin is a physiologically inert substance, i.e. it is freely filtered by the kidneys without any absorption or secretion by the tubules [7]. In clinical practice, the measurement of GFR by inulin clearance is not practical as this process is elaborate, time-consuming and expensive. It requires a continuous infusion of inulin and timed urine and serum samples. Due to these practical difficulties, studies have investigated the use of radiolabelled substances including (^51^Cr) EDTA and contrast media [8], to measure GFR precisely from a single injection. Although these agents provide measurements of GFR comparable to inulin clearance, they carry other practical difficulties. For example, radiolabelled substances require specialist licensing, handling and disposal of waste. Also, for contrast agents, the lack of robust assay technique and the large volume of contrast required have made these methods less favourable [9].

More recently iohexol, a non-ionic contrast agent, has been used to measure GFR via a single intravenous bolus followed by blood sampling 2–10 h afterwards. A study by Krutzén et al. [10] showed a correlation coefficient of 0.98 (*p* < 0.001) when iohexol was compared with ^51^Cr EDTA to measure GFR. In another study comparing iohexol with inulin clearance, there was a similarly high correlation (*r* = 0.996, *p* < 0.001) [9]. For this reason, iohexol is now widely used as an alternative gold standard to measure true GFR due to its simplicity and reliability.

## Serum creatinine and creatinine clearance

Historically, serum creatinine has been used as a surrogate of GFR based on the assumption that it is produced, filtered and secreted in a steady state. The Cockcroft–Gault (CG) equation was then developed to estimate creatinine clearance, on the presumption that creatinine clearance was a direct measure of GFR, which it is not. CG uses serum creatinine adjusted for age, weight, serum creatinine and gender to derive creatinine clearance. CG was the most widely used index of renal function [5] from its introduction in 1976 until the development of the MDRD equation, described below. CG has been validated against measured GFR using ^125^I-iothalamate, which showed that CG equation overestimated GFR by 16% [11].

The reason for this and the key limitations of using creatinine clearance and serum creatinine as estimates of GFR are that there are actually many factors which can affect the metabolism of creatinine from creatine in the muscles and the rate of secretion of creatinine in the tubules. Most clearly, creatinine is affected by muscle mass, which in turn changes with older age, and also between genders and ethnic groups. Other factors which can affect the production and secretion of creatinine and which can also differ in older versus younger patients include dietary protein intake, malnutrition and prescribed medication. Studies have also shown that creatinine clearance overestimates GFR due to the secretion of creatinine from the tubules in normal individuals. In patients with CKD, there is increased extra-renal and decreased urinary elimination of creatinine leading to overestimation of GFR from serum creatinine.

**Fig. 1:**
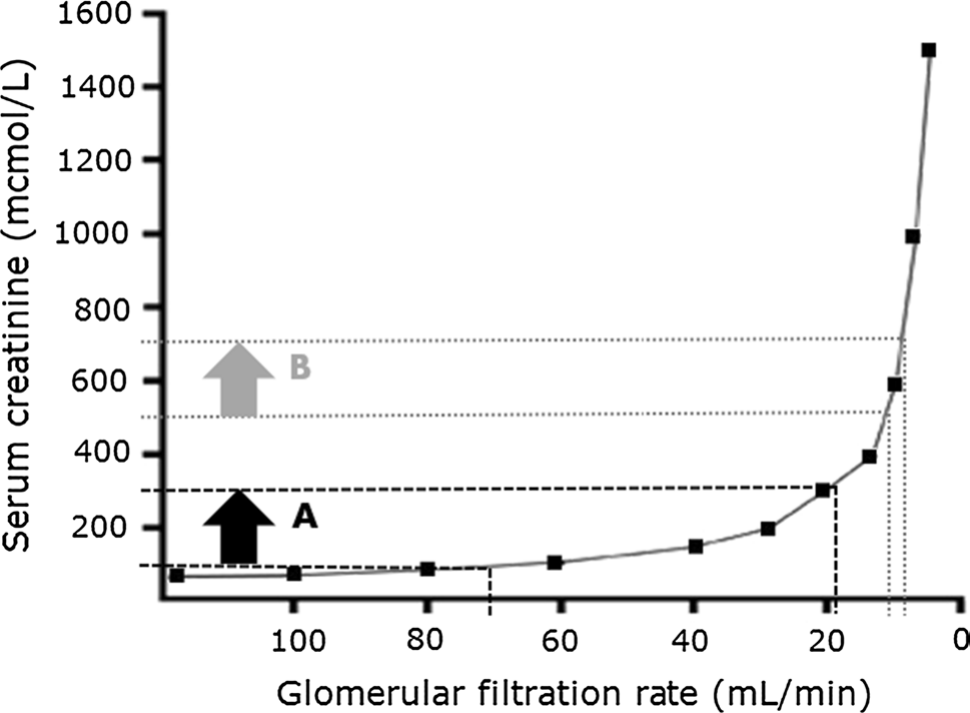
The nonlinear relationship between serum creatinine and GFR. This figure compares an increase in creatinine of 200 µmol/L (2.26 mg/dL) from two start points in a 70-year-old male. An increase in creatinine from 100 to 300 µmol/L (1.13–3.39 mg/dL) (*A*) equates to a drop in GFR of 51 mL/min (from 70 to 19 mL/min), whereas an increase in creatinine from 300 to 500 µmol/L (3.39–5.65 mg/dL) (*B*) equates to a GFR drop of only 4 mL/min (from 11 to 7 mL/min). This demonstrates that numerically similar changes in creatinine indicate far greater deterioration in kidney function for patients with better-preserved renal function at baseline

A further complicating factor in using serum creatinine alone is the nonlinear relationship between creatinine and GFR [12]. Historically, nephrologists would interpret an arbitrary percentage change in creatinine as an indicator of true change in renal function rather than reflecting day-to-day variation from factors such as dietary protein. For example, “normal” day-to-day variation in creatinine was anecdotally considered to be <10% and change beyond that was considered abnormal. Similarly, studies have used absolute change in creatinine as a marker of risk of adverse events. A prominent example is from the POSH study (prospective outcomes study in heart failure) where a rise in serum creatinine of >26 µmol/L (0.29 mg/dL) after hospitalisation for heart failure in a population with mean age 68 years was shown to increase median length of stay by 2 days (from 9 to 11 days) [13]. However, as demonstrated in Fig. 1, the same absolute change in serum creatinine can indicate markedly different changes in actual renal function measured by GFR, depending on the baseline function. In the example given in Fig. 1, a rise in serum creatinine of 200 µmol/L (2.26 mg/dL) in a 70-year-old man equates to an actual fall in GFR of 51 mL/min (from 70 to 19 mL/min) if the baseline creatinine is 100 µmol/L (1.13 mg/dL). This contrasts markedly with the same increase in creatinine of 200 µmol/L (2.26 mg/dL) from 300 to 500 µmol/L (3.39–5.65 mg/dL), which equates to a GFR drop of only 4 mL/min (from 11 to 7 mL/min). This demonstrates that numerically similar changes in creatinine indicate far greater deterioration in kidney function for patients with better-preserved renal function at baseline, and why eGFR is significantly preferred over creatinine and creatinine clearance.

## Serum creatinine-derived estimates of glomerular filtration rate

The most widely used equation for eGFR is the four-variable abbreviated MDRD equation (modification of diet in renal disease). This was adopted by the Kidney Disease Outcomes Quality Initiative (KDOQI) Clinical Practice Guidelines for Chronic Kidney Disease (CKD) [14]. The MDRD equation was initially developed from the MDRD study training sample of 1070 subjects [11]. Seven equations were developed using a regression model to predict GFR. Out of the seven equations, one containing six variables (age, sex, ethnicity, creatinine, urea and albumin) was chosen as it had an *R* ^2^ (percentage of variability in the log GFR explained by the regression model) of 90.3% on internal validation and was considered the most accurate equation to estimate GFR. Subsequently, a four-variable MDRD equation (age, sex, ethnicity, gender) was introduced to simplify the estimation of GFR [6], and in 2005, it was re-expressed to estimate GFR using isotope dilution mass spectrometry (IDMS)-traceable serum creatinine [15]. As well as measuring eGFR and not creatinine clearance, the advantage of MDRD over CG is that it does not require body weight and it was validated against actual measured GFR (calculated using ^125^I-iothalomate). The MDRD equation can easily calculate eGFR using simple demographic data and a readily available laboratory biomarker. This inclusion of ethnicity and exclusion of weight in estimating renal function were a significant departure from historical equations including CG. A summary of current and historical equations and the variables they use is found in Table 1.

**Table 1:** Variables used in current and historical equations for estimation of glomerular filtration rate from serum creatinine

Equation	Year	Variables used
FAS [25]	2016	Age, sex, S_cr_/*Q*
BIS1 [21]	2012	Age, sex, S_cr_
CKI-EPI [17]	2009	Age, sex, S_cr_, ethnicity
MDRD serum variable [11]	1999	Age, sex, S_cr_, ethnicity
Bjornsson [43]	1983	Age, sex, S_cr_, weight
Hull [44]	1982	Age, sex, S_cr_, weight
Gates [45]	1982	Age, sex, S_cr_
Cockcroft–Gault [5]	1976	Age, sex, S_cr_, weight
Jellifie [46]	1973	Age, sex, S_cr_
Mawer [47]	1972	Age, sex, S_cr_, weight
Jellifie [48]	1971	Sex, S_cr_
Reciprocal serum creatinine	–	S_cr_

*S*_*cr*_ serum creatinine, *Q* median S_cr_ from healthy population

There are, however, several limitations to the MDRD equation as it was developed and internally validated in a limited CKD population [11]. Primarily, from the perspective of this review, the MDRD study population did not include anyone over the age of 70 years. It also excluded patients with normal kidney function, and the proportion of diabetics (6%) was also small.

The performance of the MDRD equation has subsequently been externally evaluated in a diverse population of 5504 subjects from pooled data of 10 different studies [16]. In this assessment, the accuracy of the equation was assessed by the percentage of estimates that fell within 30% of measured GFR (P_30_). The overall P_30_ was 83%, and for stages 3, 4 and 5 CKD this was 84, 81, and 72%, respectively (for details of the CKD stages based on eGFR, see Table 2). In preserved renal function (eGFR levels > 60 mL/min/1.73 m^2^), the P_30_ was 84%. In the subgroup of patients aged >65 years, the P_30_ for eGFR<60 ml/min/1.73 m^2^ was 82%, and for eGFR >60 mL/min/1.73 m^2^ it was 88%. Perhaps surprisingly, by this measure MDRD performed best in older patients with preserved renal function, a subgroup specifically excluded from the original MDRD cohort.

**Table 2:** 2012 KDIGO chronic kidney disease (CKD) categories based on eGFR [35]

CKD category	eGFR (ml/min/1.73 m^2^)	Term
G1	>90	Normal
G2	60–89	Mildly decreased
G3a	45–59	Mild to moderately decreased
G3b	30–44	Moderate to severely decreased
G4	15–29	Severely decreased
G5	<15	Kidney failure

A more recent and more accurate creatinine-based method of estimating eGFR using the same four variables as MDRD has been developed, the CKD-EPI equation (Chronic Kidney Disease Epidemiology Collaboration) [17]. This has been adopted by KDIGO since 2012 but is not yet universal in clinical practice. This equation was developed from a more diverse population than MDRD, consisting of 8254 subjects pooled from 10 studies [17]. In this cohort, 15% of the population was aged >65 years, and 28% were diabetics. It also underwent external validation in 3896 subjects pooled from 16 other studies [18].

The performance of CKD-EPI and MDRD was then compared using this external validation cohort [19]. In this analysis, eGFR equation bias was expressed as the difference between measured GFR (mGFR) and eGFR, with positive values indicating underestimation of mGFR and a negative value indicating overestimation. In this study as the data were pooled from 16 different studies, the measured GFR was calculated using different exogenous filtration markers, which was one of the inclusion criteria. For an eGFR level between 16 and 29 ml/min/1.73 m^2^, the bias compared to mGFR for CKD-EPI and MDRD was similar, 1.9 and 2.0, respectively. However, the bias was better with CKD-EPI for eGFR levels above this (for example, 4.2 vs. 11.9, respectively, for an eGFR of 60–89 ml/min/1.73 m^2^).

In patients aged >65 years, the overall bias was 1.3 and 1.4 for the CKD-EPI and MDRD equations, respectively. In patients aged >65 years and with eGFR <60 ml/min/1.73 m^2^, the bias was comparable between equations (1.3 and 0.9). However, CKD-EPI performed better in patients aged >65 years with an eGFR between 60 and 89 ml/min/1.73 m^2^ (0.7 vs. 4.5).

In an independent evaluation of the performance of CKD-EPI and MDRD in older patients with CKD, old people again being defined as those aged >65 years, Kilbride et al. [20] compared the eGFR derived from these equations with mGFR calculated using iohexol. In this study, both equations overestimated mGFR, although the bias was small and was comparable (−4.3 and −5.5 for CKD-EPI and MDRD, respectively, at mGFR ≥ 60 ml/min/1.73)

However, one of the limitations of these analyses is the definition of old people, as, for example, patients aged between 65 and 70 years are likely to have significantly different physiology to those aged over 80 years. Hence, an equation aimed at estimating GFR more accurately specifically in older people has been produced by the Berlin Initiative Study (BIS) [21]. Here, all subjects were aged >70 years, and mean age was 78.5 years. The BIS1 equation, like MDRD and CKD-EPI, is creatinine based and accommodates age and gender but does not account for ethnicity.

In this study, iohexol was used to assess measured GFR. The BIS investigators compared the bias of BIS1 with MDRD and CKD-EPI equations. All overestimated mGFR but with BIS1 performing markedly better. The bias for MDRD, CKD-EPI and BIS1 was −11.2, −9.7 and −0.8, respectively. The P_30_ values were 71, 78 and 95%. A number of other studies have also compared bias and P_30_ between MDRD, CKD-EPI and BIS1 in older patients. Lopes et al. [22] compared the performance of creatinine-based BIS1, CKD-EPI and the four-variable MDRD equation against measured GFR by iohexol method in a very old population with a mean age of 85 years. In this study, BIS1 underestimated mGFR with a bias of 6.6, but CKD-EPI and MDRD both overestimated mGFR, with a bias of −1.7 and −4.6, respectively. The P_30_ was 80, 75 and 70%, respectively, for these equations. In a slightly younger population (mean age of 75 years), Koppe et al. [23] compared the performance of these equations against mGFR calculated by inulin. In this study, the bias was small and comparable with all three equations overestimating mGFR with a bias of −4.1, −5.4 and −5.8 for BIS, CKP-EPI and MDRD equations, respectively, and P_30_ of 76, 72 and 71%, respectively. Liu et al. [24] also compared the performance of these equations, but in this case in a Chinese population and one with a mean age of 70 years. mGFR was measured by ^99m^Tc-DTPA. In this study, the bias was again small (−0.31, −1.88 and 1.78 for BIS, CKD-EPI and MDRD, respectively), but the P_30_ was very low compared to the studies listed above (63, 55 and 55%, respectively). Pottel et al. developed an eGFR equation for people of all ages (FAS). This used serum creatinine derived from the healthy population, adjusted for age and sex. The performance of this equation was validated using a pooled data set of 6870 subjects of various age groups (<18, 18–70 and ≥70 years). The mGFR was calculated using various methods of clearance: inulin, iohexol and iothalamate. In the age group ≥70 years, the FAS equation performance was superior to CKD-EPI with a bias of 1.1 and −5.6, respectively, and a P_30_ of 86.1 and 77.6, respectively [25]. These studies, and others which have compared BIS1 and CKD-EPI only [26, 27], are summarised in Table 3. What is perhaps most noteworthy is that the markedly better performance of BIS1 in the study by the BIS investigators has not been matched in other studies.

**Table 3:** Comparisons of performance of creatinine-derived eGFR equations in elderly patients from different study groups

References	Age (years)	*N*	mGFR method	mGFR (ml/min/1.73 m^2^)	eGFR equation	Bias	P_30_	Best performance
Pottel et al. [25]	70^c^	1764	Various^d^	55.6	FAS	1.1	86.1	FAS
					CKD-EPI	−5.6	77.6	
Fan et al. [26]	80^a^	805	Iohexol	62 ± 17	BIS1	5.7	95.8	BIS1
					CKD-EPI	−2.7	91.7	
Alshaer et al. [27]	80^b^	394	Iohexol	53 (7–101)	BIS1	3.6	88	BIS1
					CKD-EPI	−2.3	83	
Kilbride et al. [20]	80^b^	394	Iohexol	53 (7–101)	MDRD	−3.5	81	CKD-EPI
					CKD-EPI	−1.7	83	
Schaeffner et al. [21]	79^a^	285	Iohexol	60 (16–117)	BIS1	−0.8	95.1	BIS1
					MDRD	−11.2	70.9	
					CKD-EPI	−9.6	77.9	
Stevens et al. [19]	71^b^	476	Various^d^	<60	MDRD	3.3	–	MDRD
					CKD-EPI	4.3	–	
		85		60–89	MDRD	5.9	–	CKD-EPI
					CKD-EPI	1.0	–	
Lopes et al. [22]	85^a^	56	Iohexol	<60	BIS1	1.9	80	BIS1
					CKD-EPI	−3.6	75	
					MDRD	−5.9	71	
		39		≥60	BIS1	13.4	82	CKD-EPI
					CKD-EPI	1.0	90	
					MDRD	−2.7	80	
Koppe et al. [23]	75^a^	224	Inulin	41 ± 17	BIS1	−4.1	76	BIS1
					MDRD	−5.8	71	
					CKD-EPI	−5.4	72	
Liu et al. [24]	70^a^	332	^99m^Tc-DTPA	40 (24–117)	MDRD	1.78	55	BIS1
					CKD-EPI	−1.88	55	
					BIS1	−0.31	63	

*MDRD* modification of diet in renal disease, *CKD-EPI* Chronic Kidney Disease Epidemiology Collaboration, *BIS* Berlin initiative study *P*_*30*_ percentage of eGFR within 30% of the measured GFR (accuracy), *eGFR* estimated glomerular filtration rate, *FAS* full age spectrum

^a^ Mean age

^b^ Median age

^c^ Age over 70 years

^d^ Measured GFR (mGFR) data pooled from various studies, which could have used different exogenous filtration markers for the measurement of mGFR, bias calculated by the mean difference between mGFR and eGFR (mGFR-eGFR) hence a negative value would mean overestimation and a positive value mean underestimation of GFR compared to the measured GFR

## Cystatin C

The limitations of creatinine discussed above have also led to the development of eGFR equations based on other serum biomarkers. One such biomarker which has now making its way into routine clinical care is cystatin C. Cystatin C is a protease inhibitor that is freely filtered through the glomeruli, reabsorbed and degraded by the proximal tubules (a comparison of the renal handling of common biomarkers used in GFR measurement and estimation is found in Table 4). Importantly, current evidence indicates that cystatin C levels are not affected by the age or muscle mass of an individual [28, 29]. Fliser et al. demonstrated this is in a study that compared mGFR measured by inulin clearance in older versus younger patients (mean ages 67 vs. 25 years) with identical plasma creatinine (mean of 81 µmol/L [0.92 mg/dL] in both groups, *p* = 0.90). Despite the identical creatinine, mGFR was modestly but statistically significantly lower in the older group (104 ± 12 mL/min/1.73 m^2^ vs. 119 ± 11 mL/min/1.73 m^2^, *p* < 0.001). The mean serum cystatin C was significantly higher in the older age group (0.84 ± 0.10 mg/L vs. 0.69 ± 0.08 mg/L, *p* < 0.001). There was better correlation between serum cystatin C and inulin clearance (*r* = −0.65; *p* < 0.001) than serum creatinine and plasma inulin clearance (*r* = −0.30; *p* < 0.02) This suggests that cystatin C is superior to creatinine for estimating GFR in the elderly [30].

**Table 4:** Properties of the most commonly used biomarkers for measuring or estimating glomerular filtration rate

Filtration marker	Source	Type	Filtration property
Creatinine	Endogenous	Metabolised from creatine	Freely filtered through the glomeruli
			Secreted from the proximal tubule
			Extra-renal elimination (stools/sweat)
Cystatin	Endogenous	Proteinase inhibitor	Stable plasma concentration
			Freely filtered through glomeruli
			Reabsorbed and metabolised in the proximal tubule
Inulin	Exogenous	Fructose polysaccharide	Physiologically inert
			Freely filtered through the glomeruli
			Not absorbed, secreted, synthesised or metabolised in the kidney
			Stable plasma concentration
Iohexol	Exogenous	Non-ionic water soluble contrast medium	Freely filtered through glomeruli
			Not secreted or reabsorbed in the tubules

An equation using cystatin C has been developed by the CKD-EPI, Caucasian Asian Paediatric Adult (CAPA) and Japanese study groups. In a comparison of performance against mGFR in 805 older patients with a mean age of 80 years, the P_30_ values were comparable between equations (94, 94, 93%, respectively). The CAPA equation had negligible bias (0.1) and CKD-EPI_cystatin_, and the Japanese equation both modestly underestimated mGFR (Table 5) [26].

**Table 5:** Comparisons of performance of cystatin C derived eGFR equations in elderly patients from different study groups

References	Age (years)	*N*	mGFR method	mGFR (ml/min/1.73 m^2^)	eGFR equation	Bias	P_30_	Best performance
Fan et al. [26]	80^a^	805	Iohexol	62 ± 17	CKD-EPI	1.9	93.8	CAPA
					Japanese	4.6	92.8	
					CAPA	0.1	94.4	
Alshaer et al. [27]	80^b^	394	Iohexol	53 (7–101)	CKD-EPI	0.1	86	–
Kilbride et al. [20]	80^b^	234	Iohexol	<60	CKD-EPI^d^	−3.4	91.0	–
		160		≥60	CKD-EPI^e^	2.9	82.0	–
Schaeffner et al. [21]	79^a^	285	Iohexol	60 (16–117)	CKD-EPI	−2.05	89.1	–

*CKD-EPI* chronic kidney disease epidemiology collaboration, *CAPA* Caucasian, Asian, paediatric, and adult cohort, *IQR* interquartile range (precision), *P*_*30*_ percentage of eGFR within 30% of the measured GFR (accuracy), *eGFR* estimated glomerular filtration rate

^a^ Mean age

^b^ Median age

^c^ Measured GFR (mGFR) data pooled from various studies, which could have used different exogenous filtration markers for the measurement of mGFR, bias calculated by the mean difference between mGFR and eGFR (eGFR-mGFR); hence, a negative value would mean overestimation and a positive value mean underestimation of GFR compared to the measured GFR

^d^ Normal mGFR indicates group with measured GFR of ≥60 mL/min/1.73 m^2^

^e^ CKD indicates group with measured GFR <60 mL/min/1.73 m^2^

As a development from equations using creatinine or cystatin C alone, many of the study groups listed above have derived eGFR equations using both creatinine and cystatin C in the same model. Such models appear to be more accurate than using either biomarker alone. For example, one study has compared CKD-EPI equations using creatinine alone, cystatin C alone, and both in 95 patients aged 85 ± 4 years (mean mGFR 55 ± 15 mL/min/1.73 m^2^). The respective P30 values were 75, 65 and 85%. The respective bias values were −1.7, 7.4 and 4.0 [22]. Of the combined creatinine/cystatin C equations, the one produced by the BIS team, BIS2, appears to consistently perform the best (Table 6). Although the use cystatin C improves the accuracy of an equation, it is not readily available in all clinical laboratories and its measurement is more expensive than that of creatinine. However, these factors should not discourage the use of cystatin C in estimating GFR.

**Table 6:** Comparisons of performance of eGFR equations which use both creatinine and cystatin C in elderly patients from different study groups

References	Age (years)	*N*	mGFR method	mGFR (ml/min/1.73 m^2^)	eGFR equation	Bias	P_30_	Best performance
Fan et al. [26]	80^a^	805	Iohexol	62 ± 17	CKD-EPI	−0.6	96.1	BIS2
					Japanese	8.2	93	
					BIS2	5.3	97.9	
Alshaer et al. [27]	80^b^	394	Iohexol	53 (7–101)	BIS2 CKD-EPI	−1.2	86	
Kilbride et al. [20]	80^b^	394	Iohexol	53 (7–101)	CKD-EPI	−1.2	86	–
Schaeffner et al. [21]	79^a^	285	Iohexol	60 (16–117)	BIS2 CKD-EPI	−9.22	81.4	
Lopes et al. [22]	85^a^	95	Iohexol	55 (19–86)	BIS2 CKD-EPI	4.0	85	

*CKD-EPI* Chronic Kidney Disease Epidemiology Collaboration, *CAPA* Caucasian, Asian, paediatric, and adult cohort, *P*_*30*_ percentage of eGFR within 30% of the measured GFR (Accuracy), *eGFR* estimated glomerular filtration rate

^a^ Mean age

^b^ Median age

^c^ Measured GFR (mGFR) data pooled from various studies, which could have used different exogenous filtration markers for the measurement of mGFR, bias calculated by the mean difference between mGFR and eGFR (mGFR-eGFR); hence, a negative value would mean overestimation and a positive value mean underestimation of GFR compared to the measured GFR

## Measuring renal function in AKI

Older people are susceptible to AKI due to age-related loss of glomeruli and glomerular capillaries, dysfunction of the vascular auto-regulatory system and tubular frailty. Hence, urinary and serum biomarkers are likely to be less reliable in older people [31]. It is vital to note that the creatinine and cystatin C methods of estimating GFR discussed here are not validated for use in acute kidney injury (AKI). The test and validation cohorts, and therefore the equations, all presume a steady state of renal function. The rise in serum creatinine associated with AKI typically lags behind the actual kidney injury, possibly not indicating AKI until >24 h after the precipitating event. This not only precludes the use of eGFR equations in AKI but also actually limits the use of change in serum creatinine as a biomarker in AKI. An ideal biomarker for AKI will present an abnormal value earlier than change in creatinine. In addition, in conditions such as sepsis, which are associated with AKI, animal models have shown that creatinine production may be decreased, further limiting the detection of AKI with this biomarker.

An additional reason why no novel biomarker has emerged for AKI in the way that cystatin C has for CKD may be that evaluating a new biomarker requires a suitable gold standard. Given that the current gold standard is limited to change in serum creatinine, the accuracy of new tools is difficult to ascertain. Instead, grading of AKI in such a way as to accurately risk stratify patients has emerged in place of a single laboratory biomarker. It can be argued that the clinical requirement of an effective AKI biomarker is that it can improve management, and this does not necessarily mean that a very precise GFR measurement is needed; to know that AKI has occurred and to apply relevant management strategies is the goal.

The emerging increased emphasis on early detection and management of AKI is because of the association with increased mortality, morbidity and hospital length of say [32–36]. In a meta-analysis of 48 studies including 47,017 participants looking at the long-term outcomes following AKI [32], the incidence of mortality, CKD and ESRD was 8.9, 7.8 and 4.9 events per 100 patient years, respectively. This increased risk is vastly greater in elderly patients. For example, in a community-based study, the incidence of AKI requiring dialysis for subjects aged of 70–79 years was 96 per 100,000 patient years. This compared to just 8 per 100,000 in subjects aged <50 years. Also, in the older age group, the incidence increased from 74 to 123 events per 100 000 patient years between the years 1996 and 2003 [33].

The current KDIGO guidelines define AKI as any of the following [34]: an increase in serum creatinine of ≥26.5 µmol/L (0.29 mg/dL) within 48 h; an increase in serum creatinine to 1.5 times the previous baseline which is known or presumed to have occurred within the previous 7 days; or urine output of <0.5 ml/kg/h for 6 h. These definitions do not stratify patients by risk. The current stages of AKI based on serum creatinine by which risk and management are stratified are as follows:

Stage 1: 1.5–1.9 times baseline OR ≥ 26.5 µmol/L (0.29 mg/dL) increase;
Stage 2: 2.0–2.9 times baseline;
Stage 3: ≥3.0 times baseline OR serum creatinine >350 µmol/L (3.95 mg/dL) or dialysis.

## Current clinical practice

The 2012 KDIGO clinical practice guidelines have recommended the use of the creatinine-derived CKD-EPI equation for the estimation of eGFR in routine practice. This is because cystatin C is not yet available for daily use in all centres. The guidelines suggest that cystatin C-based equations be limited to use in individuals with eGFR between 45 and 59 ml/min/1.73 m^2^ with no other evidence of CKD [35] to verify the presence of CKD. There was no specific recommendation based on age, and so the apparent better performance of BIS2 in the elderly does not translate into a clinical recommendation.

Changing the recommended equation for calculation of eGFR has a consequent impact on the reported prevalence of CKD. For example, the prevalence of CKD in 754 subjects with a mean age of 61 years was 17.2% based on the four-variable MDRD equation that was recommended for use until 2012, whereas with an equation based on both creatinine and cystatin C the prevalence is as low as 5.8% [36].

## Future of GFR measurement

The conventional measurement of GFR involves the measurement of a biomarker in either urine or blood. This is invasive and time-consuming and provides no longitudinal data unless repeated. The use of transcutaneous measurement of GFR in rat models has evolved significantly. This method of GFR is non-invasive and can measure real-time GFR. Several methods of transcutaneous measurement of GFR are described and are based on the elimination kinetics of fluorescent exogenous biomarker like flourescein isothiocyanate (FITC)-sinistrin [37–41]. Recent studies have used FITC-sinistrin in the conscious rats and have validated their results against plasma measurement of FITC-sinistrin. Schreiber et al. showed that transcutaneous measurement of FITC-sinstrin against two-compartment plasma clearance and one compartment plasma slope in 3 different mouse models was comparable in healthy animals (*n* = 8). The GFR measurements (mean ± standard deviation) for each were 1381 ± 264, 1373 ± 182 and 1212 ± 274 µL/min/100 g body weight, respectively [42]. Such novel methods may shape the future of GFR measurement in humans.

## Summary

The estimation of GFR in older patients is based on equations largely derived in younger populations despite physiological changes with age that may affect metabolism of biomarkers. Despite this, the performance of eGFR equations is becoming more robust for the older population group. In particular, the use of cystatin C has the potential to improve CKD diagnosis and epidemiology, as its physiological behaviour is different to creatinine. Current guidelines do not specify the use of cystatin C-based equations as first line for calculating eGFR, but this reflects its often-limited availability rather than poor performance. Indeed, if there is any clinical suspicion that a patient’s clinical status and calculated eGFR do not match, then a combined cystatin C/creatinine equation (CKD-EPI or BIS2) is a useful next step before one needs to consider isotope GFR studies for mGFR (see also Table 7). The caveat to this is in AKI, where accurate measurement of GFR at a specific time point is not yet viable, nor indeed is it necessarily useful. Instead, management guidelines for AKI are based on the KDIGO AKI stages, which can be determined from change in serum creatinine. Transcutaneous measurement of GFR may hold the key to the future of non-invasive and real-time measurement of GFR in various clinical settings including AKI.

**Table 7:** Key points

eGFR is influenced by several factors including age
eGFR equations are only validated for use in CKD, not AKI
2012 KDOGI guidelines recommend the use of CKD-EPI equation to calculate eGFR instead of the four-variable MDRD equation due to better accuracy
Cystatin C in combination with creatinine appears to be superior in estimating GFR and needs to be considered in special circumstances
BIS equations appear to be the more accurate in estimating GFR in the elderly but there is not yet any recommendation for an eGFR equation specific to this age group

## References

[1] Zhou XJ, Rakheja D, Yu X, Saxena R, Vaziri ND, Silva FG (2008). The aging kidney. Kidney Int.

[2] Glassock RJ, Winearls C (2009). Ageing and the glomerular filtration rate: truths and consequences. Trans Am Clin Climatol Assoc.

[3] Davies DSN (1950). Age change in glomerular filteration rate, effective renal plasma flow and tubular. Clin Invest.

[4] Garg A, Papaioannou A, Ferko N (2004). Estimating the prevalence of renal insufficiency in seniors requiring long-term care. Kidney.

[5] Cockcroft DW, Gault MH (1976). Prediction of creatinine clearance from serum creatinine. Nephron.

[6] Levey AS (2000). A simplified equation to predict glomerular filtration rate from serum creatinine. J Am Soc Nephrol.

[7] Schuster VL, Seldin DW (1992). Renal clearance. Kidney Physiol Pathophysiol.

[8] Bäck S-E, Krutzén E, Nilsson-Ehle P (1988). Contrast media as markers for glomerular filtration: a pharmacokinetic comparison of four agents. Scand J Clin Lab Invest.

[9] Gaspari F, Perico N, Ruggenenti P, Mosconi L, Amuchastegui CS, Guerini E, Daina E, Remuzzi G (1995). Plasma clearance of nonradioactive iohexol as a measure of glomerular filtration rate. J Am Soc Nephrol.

[10] Krutzén E, Back S-E, Nilsson-Ehle I, Nilsson-Ehle P (1984). Plasma clearance of a new contrast agent, iohexol: a method for the assessment of glomerular filtration rate. J Lab Clin Med.

[11] Levey A, Bosch J, Lewis J (1999). A more accurate method to estimate glomerular filtration rate from serum creatinine; a new prediction equation. Ann Intern.

[12] Shemesh O, Golbetz H, Kriss JP, Myers BD (1985). Limitations of creatinine as a filtration marker in glomerulopathic patients. Kidney Int.

[13] Cowie MR (2005). Prevalence and impact of worsening renal function in patients hospitalized with decompensated heart failure: results of the prospective outcomes study in heart failure (POSH). Eur Heart J.

[14] National Kidney Foundation (2002) K/DOQI clinical practice guidelines for chronic kidney disease: evaluation, clasification and stratification, vol 39 11904577

[15] Levey AS, Coresh J, Greene T, Marsh J, Stevens LA, Kusek J, Van Lente F (2005). Expressing the MDRD study equation for estimating GFR with IDMS traceable (gold standard) serum creatinine values. J Am Soc Nephrol.

[16] Stevens LA, Coresh J, Feldman HI, Greene T, Lash JP, Nelson RG, Rahman M, Deysher AE, Zhang YL, Schmid CH, Levey AS (2007). Evaluation of the modification of diet in renal disease study equation in a large diverse population. J Am Soc Nephrol.

[17] Levey AS, Stevens LA, Schmid CH, Zhang Y, Castro Alejandro F, Feldman HI, Kusek JW, Eggers P, Van Lente F, Greene T, Coresh J (2009). A new equation to estimate glomerular filtration rate. Ann. Intern. Med..

[18] Stevens LA, Schmid CH, Zhang YL, Coresh J, Manzi J, Landis R, Bakoush O, Contreras G, Genuth S, Klintmalm GB, Poggio E, Rossing P, Rule AD, Weir MR, Kusek J, Greene T, Levey AS (2010). Development and validation of GFR-estimating equations using diabetes, transplant and weight. Nephrol Dial Transplant.

[19] Stevens LA, Schmid CH, Greene T, Zhang YL, Beck GJ, Froissart M, Hamm LL, Lewis JB, Mauer M, Navis GJ, Steffes MW, Eggers PW, Coresh J, Levey AS (2010). Comparative performance of the CKD Epidemiology Collaboration (CKD-EPI) and the Modification of Diet in Renal Disease (MDRD) Study equations for estimating GFR levels above 60 mL/min/1.73 m2. Am J Kidney Dis.

[20] Kilbride HS, Stevens PE, Eaglestone G, Knight S, Carter JL, Delaney MP, Farmer CKT, Irving J, O’Riordan SE, Dalton RN, Lamb EJ (2013). Accuracy of the MDRD (modification of diet in renal disease) study and CKD-EPI (CKD epidemiology collaboration) equations for estimation of GFR in the elderly. Am J Kidney Dis.

[21] Schaeffner ES, Ebert N, Delanaye P, Frei U, Gaedeke J, Jakob O, Kuhlmann MK (2012). Two novel equations to estimate kidney function in persons aged 70 years or older. Ann Intern Med.

[22] Lopes MB, Araújo LQ, Passos MT, Nishida SK, Kirsztajn GM, Cendoroglo MS, Sesso RC (2013). Estimation of glomerular filtration rate from serum creatinine and cystatin C in octogenarians and nonagenarians. BMC Nephrol.

[23] Koppe L, Klich A, Dubourg L, Ecochard R, Hadj-Aissa A (2013). Performance of creatinine-based equations compared in older patients. J Nephrol.

[24] Liu X, Chen J, Wang C, Shi C, Cheng C, Tang H, Lou T (2013). Assessment of glomerular filtration rate in elderly patients with chronic kidney disease. Int Urol Nephrol.

[25] Pottel H, Hoste L, Dubourg L, Ebert N, Schaeffner E, Eriksen BO, Melsom T, Lamb EJ, Rule AD, Turner ST, Glassock RJ, De Souza V, Selistre L, Mariat C, Martens F, Delanaye P (2016). An estimated glomerular filtration rate equation for the full age spectrum. Nephrol Dial Transplant.

[26] Fan L, Levey AS, Gudnason V, Eiriksdottir G, Andresdottir MB, Gudmundsdottir H, Indridason OS, Palsson R, Mitchell G, Inker LA (2015). Comparing GFR estimating equations using cystatin C and creatinine in elderly individuals. J Am Soc Nephrol.

[27] Alshaer IM, Kilbride HS, Stevens PE, Eaglestone G, Knight S, Carter JL, Delaney MP, Farmer CKT, Irving J, O’Riordan SE, Dalton RN, Lamb EJ (2014). External validation of the Berlin equations for estimation of GFR in the elderly. Am J Kidney Dis.

[28] Grubb AO (2000). Cystatin C–properties and use as diagnostic marker. Adv Clin Chem.

[29] Newman DJ (2002). Cystatin C. Ann Clin Biochem.

[30] Fliser D, Ritz E (2001). Serum cystatin C concentration as a marker of renal dysfunction in the elderly. Am J Kidney Dis.

[31] Musso CG, Liakopoulos V, Ioannidis I, Eleftheriadis T, Stefanidis I (2006). Acute renal failure in the elderly: particular characteristics. Int Urol Nephrol.

[32] Coca SG, Yusuf B, Shlipak MG, Garg AX, Parikh CR (2009). Long-term risk of mortality and other adverse outcomes after acute kidney injury: a systematic review and meta-analysis. Am J Kidney Dis.

[33] Hsu C-Y, McCulloch CE, Fan D, Ordoñez JD, Chertow GM, Go AS (2007). Community-based incidence of acute renal failure. Kidney Int.

[34] Kellum JA, Lameire N, Aspelin P, Barsoum RS, Burdmann EA, Goldstein SL, Herzog CA, Joannidis M, Kribben A, Levey AS, MacLeod AM, Mehta RL, Murray PT, Naicker S, Opal SM, Schaefer F, Schetz M, Uchino S (2012). KDIGO clinical practice guideline for acute kidney injury. Kidney Int Suppl.

[35] Kidney Disease: Improving Global Outcomes (KDIGO) CKD Work Group (2013). KDIGO 2012 clinical practice guideline for the evaluation and management of chronic kidney disease. Kidney Int Suppl.

[36] Delanaye P, Cavalier E, Saint-Remy A, Lutteri L, Krzesinski J-M (2009). Discrepancies between creatinine-based and cystatin C-based equations in estimating prevalence of stage 3 chronic kidney disease in an elderly population. Scand J Clin Lab Invest.

[37] Schock-Kusch D, Xie Q, Shulhevich Y, Hesser J, Stsepankou D, Sadick M, Koenig S, Hoecklin F, Pill J, Gretz N (2011). Transcutaneous assessment of renal function in conscious rats with a device for measuring FITC-sinistrin disappearance curves. Kidney Int.

[38] Yu W, Sandoval RM, Molitoris BA (2007). Rapid determination of renal filtration function using an optical ratiometric imaging approach. Am J Physiol Renal Physiol.

[39] Schock-Kusch D, Sadick M, Henninger N, Kraenzlin B, Claus G, Kloetzer HM, Weiß C, Pill J, Gretz N (2009). Transcutaneous measurement of glomerular filtration rate using FITC-sinistrin in rats. Nephrol Dial Transplant.

[40] Pill J, Kraenzlin B, Jander J, Sattelkau T, Sadick M, Kloetzer H-M, Deus C, Kraemer U, Gretz N (2005). Fluorescein-labeled sinistrin as marker of glomerular filtration rate. Eur J Med Chem.

[41] Poreddy AR, Neumann WL, Freskos JN, Rajagopalan R, Asmelash B, Gaston KR, Fitch RM, Galen KP, Shieh J-J, Dorshow RB (2012). Exogenous fluorescent tracer agents based on pegylated pyrazine dyes for real-time point-of-care measurement of glomerular filtration rate. Bioorg Med Chem.

[42] Schreiber A, Shulhevich Y, Geraci S, Hesser J, Stsepankou D, Neudecker S, Koenig S, Heinrich R, Hoecklin F, Pill J, Friedemann J, Schweda F, Gretz N, Schock-Kusch D (2012). Transcutaneous measurement of renal function in conscious mice. Am J Physiol Renal Physiol.

[43] Bjornsson TD, Cocchetto DM, McGowan FX, Verghese CP, Sedor F (1983). Nomogram for estimating creatinine clearance. Clin Pharmacokinet.

[44] Hull JH, Hak LJ, Koch GG, Wargin WA, Chi SL, Mattocks AM (1981). Influence of range of renal function and liver disease on predictability of creatinine clearance. Clin Pharmacol Ther.

[45] Gates GF (1985). Creatinine clearance estimation from serum creatinine values: an analysis of three mathematical models of glomerular function. Am J Kidney Dis.

[46] Jelliffe RW (1973). Letter: creatinine clearance: bedside estimate. Ann Intern Med.

[47] Mawer GE, Knowles BR, Lucas SB, Stirland RM, Tooth JA (1972). Computer-assisted prescribing of kanamycin for patients with renal insufficiency. Lancet.

[48] Jelliffe R (1971). Estimation of creatinine clearance when urine cannot be collected. Lancet.

